# Maternal Vitamin and Mineral Supplementation Affected Neonatal Gene Expression and Rewired Key Regulatory Genes Underlying Hepatic Metabolism

**DOI:** 10.3390/ani15182664

**Published:** 2025-09-11

**Authors:** Audrey J. Craner, Carl R. Dahlen, Jennifer L. Hurlbert, Ana Clara B. Menezes, Priyanka Banerjee, Friederike Baumgaertner, Kerri A. Bochantin-Winders, Samat Amat, Kevin K. Sedivec, Kendall C. Swanson, Wellison J. S. Diniz

**Affiliations:** 1 Department of Animal Sciences, Auburn University, Auburn, AL 36849, USA; ajc0097@auburn.edu (A.J.C.); pzb0035@auburn.edu (P.B.); 2 Department of Animal Sciences, Center for Nutrition and Pregnancy, North Dakota State University, Fargo, ND 58108, USA; jennifer.hurlbert@ndsu.edu (J.L.H.); friederike.baumgrtne@ndsu.edu (F.B.); kerri.bochantin@ndus.edu (K.A.B.-W.); kendall.swanson@ndsu.edu (K.C.S.); 3 Department of Animal Science, South Dakota State University, Brookings, SD 57007, USA; anaclara.baiaomenezes@sdstate.edu; 4 Department of Microbiological Sciences, North Dakota State University, Fargo, ND 58108, USA; samat.amat@ndsu.edu; 5 Central Grasslands Research Extension Center, North Dakota State University, Streeter, ND 58483, USA; kevin.sedivec@ndsu.edu

**Keywords:** beef cattle, fetal programming, liver programming, maternal mineral and vitamin supplementation, mitochondrial function, oxidative phosphorylation, transcriptome

## Abstract

We investigated gene regulatory relationships in the neonatal liver to identify potential mechanisms by which maternal vitamin and mineral supplementation during gestation influences developmental programming in heifer calves. Liver transcriptomes were profiled by RNA-Seq in calves collected 30 h after birth from dams fed either a basal diet (CON; *n* = 6) or the basal diet plus a vitamin and mineral premix (VTM; *n* = 6; 113 g·heifer^−1^·d^−1^), starting 60 days pre-breeding and continuing through gestation. Using DESeq2, we identified 630 DEGs between treatment groups (*p* ≤ 0.05 and |log2FC| ≥ 0.5). Over-represented pathways included oxidative phosphorylation, AMPK, PI3K/Akt, and FoxO, key regulators of energy homeostasis. We also identified 58 transcription factors (TFs) and 435 differentially connected genes (*p* ≤ 0.05), suggesting maternal diet induced a regulatory rewiring of TFs and histone deacetylating genes. Gene set enrichment analysis revealed genes positively ranked, such as *LRAT* and *LMBRD1*, underlying the vitamin digestion and absorption pathway, indicating coordinated upregulation in the VTM group. Conversely, 23 DEGs associated with the OXPHOS KEGG pathway were downregulated in the VTM calves. Further research should explore whether these transcriptional changes are linked to epigenetic modifications established at birth, persist throughout postnatal development, and potentially contribute to multigenerational inheritance.

## Simple Summary

Pregnancy and early life are sensitive windows of developmental plasticity that shape offspring growth and performance. Our study investigates the impacts of providing pregnant heifers with a vitamin and mineral supplement on calf outcomes at birth. Accordingly, the hepatic transcriptome was measured using RNA-Seq in 12 calves collected 30 h after calving from dams fed a diet initiated 60 days pre-breeding and continuing throughout gestation (VTM or CON, *n* = 6/group). We identified 630 differentially expressed genes (DEGs) between treatment groups. Pathways underlying energy homeostasis, such as oxidative phosphorylation, AMP-activated protein kinase (AMPK), Phosphoinositide 3-kinase/Protein Kinase B (PI3K/Akt), and Forkhead box O (FoxO), were over-represented by DEGs. Maternal vitamin and mineral supplementation throughout gestation was associated with molecular changes in the neonatal liver via transcriptomic responses. Further research is warranted to determine the mechanisms of fetal programming and potential multigenerational inheritance.

## 1. Introduction

Gestation encompasses a synchronized sequence of events essential for the developing fetus. This includes intricate processes, such as gastrulation, organogenesis, and cellular growth and differentiation, which ensure the fetus is prepared for life outside the womb [1]. Maternal nutrition is among the major factors critical for the proper development and function of fetal organs [1]. Changes in the maternal environment, including the availability of nutrients, during critical windows of development can contribute to fetal metabolic programming in adulthood and even across generations [2]. In beef production systems, due to seasonal variations, many commonly grazed forages lack sufficient levels of essential vitamins and minerals to meet the ruminant maintenance and production requirements [3]. These micronutrients play essential roles in processes such as energy metabolism, DNA synthesis, and epigenetic modulation of gene expression [4]. Additionally, growing evidence has shown their role in programming key biological processes during embryonic and fetal development [5,6,7,8]. Despite the evidence of its benefits, the adoption of vitamin and mineral supplementation strategies remains inconsistent and limited among cattle producers, with practices varying widely based on region, resources, and management priorities [5,9].

We have reported that vitamin and mineral supplementation during early gestation affects the dam’s metabolism and the available nutrients for fetal development. Changes were observed in placental development and the expression of angiogenic factors, energy metabolism, and nutrient transport-related genes at day 83 of gestation [10,11]. Likewise, amino acid, carbohydrate, and energy profiles in the maternal serum, allantoic fluid, and fetal liver were affected [5,12,13]. A more comprehensive study reported that vitamin and mineral supplementation from 60 days pre-breeding or breeding through calving did not affect calf morphometrics or organ weights at birth [8]. However, the concentrations of trace minerals (Se, Cu, Zn, and Co) in the calf liver at birth were affected, and calves born to VTM supplemented cows had greater body weight from weaning to 15 months of age than their CON counterparts [7]. These results suggest that fetal organ development undergoes adaptive changes in response to nutrient imbalances [14], which have been associated with altered gene expression and epigenetic regulation, including DNA methylation.

Formed in the early gestation, the liver serves as a central organ for coordinating metabolic functions [15]. Changes in visceral organs during pregnancy can impact the offspring’s metabolic efficiency and health. Altered hepatic programming may affect nutrient partition and lead to metabolically compromised offspring [16,17], which poses major challenges to the livestock industry by reducing production efficiency [18]. Additionally, compromised developmental programming can negatively affect selection and breeding decisions as the phenotype may not reflect the offspring’s genetic potential [19]. In previous research, fetuses from supplemented dams during the first 83 days of gestation resulted in calves with higher concentrations of Se, Cu, Zn, Mo, and Co in the fetal liver [13]. Additionally, maternal supplementation resulted in greater fetal liver weights and changes in the hepatic lipidome, metabolome, and transcriptome of energy- and lipid-related genes [6,20,21]. Likewise, growing evidence shows that vitamins and minerals influence the epigenome during embryonic development and throughout life [4]. These effects and epigenetic marks may also be inherited across generations, affecting offspring performance [16,19,22]. Despite these findings, the mechanisms by which vitamin and mineral supplementation affect liver development and programming are unknown. Furthermore, it is important to assess the short-term and lasting impacts of maternal supplementation on subsequent progeny performance.

Building on our previous research at day 83 of gestation and the gap in knowledge related to the hepatic transcriptome at birth, we hypothesized that supplementing dams with vitamins and minerals pre-breeding and continuing through gestation would alter physiological and transcriptomic responses in neonatal calves through transcription factors modulating hepatic gene expression and regulatory networks. Thus, our objective was to identify differentially expressed genes in the neonatal liver and the underlying biological processes involved in hepatic metabolism and function. Furthermore, we analyzed gene–gene interactions to identify regulatory genes responsive to maternal diet.

## 2. Materials and Methods

### 2.1. Care and Use of Animals

Animal use and experimental protocols used in this project were reviewed and approved by the North Dakota State University (NDSU, Fargo, ND, USA) Institutional Animal Care and Use Committee (#A21047/2021-03/26).

### 2.2. Animal Management and Experimental Design

We have previously published an article describing the animal model and experiment design [7,8]. In brief, the study utilized 72 crossbred Angus-based heifers (aged 14 to 15 months, with an initial body weight (BW), of 380.4 ± 50.56 kg) to investigate the impact of vitamin and mineral supplementation throughout gestation on offspring development. The heifers were first acclimated for 14-day to an individual feeding system (American Calan; Northwood, NH, USA). Then, heifers underwent estrus synchronization using the 7-day Select Synch + CIDR protocol [23]. Heifers were then artificially inseminated (AI) using female-selected semen from a single bull. Post-AI, heifers were stratified by BW and randomly allocated to one of two diets: control (CON; *n* = 36) or vitamin and mineral supplemented (VTM; *n* = 36). The VTM group received a premix supplement (113 g·heifer^−1^·d^−1^) in addition to the basal ration. Open heifers after pregnancy check were rebred 60 days after the first AI (CON, *n* = 19; VTM, *n* = 18), following the breeding protocol described above. Rebreeding was based on the treatment groups to which the heifers were initially assigned. Pregnant heifers from rebreeding were included in our study and used to describe the animal model and to characterize the jejunal mucosa transcriptome [8,24].

The VTM treatment started 60 days before breeding and continued until parturition. From the start of treatment until day 239 of gestation, the VTM group received a loose-form of premix supplement (Purina Wind and Rain Storm All Season 7.5 Complete, Land O’Lakes, Inc., Arden Hills, MN, USA; Table S2) that was top-dressed onto their basal ration. Beginning on day 240, the VTM premix was incorporated directly into the total mixed ration (TMR) for the supplemented animals. Detailed diet nutrient profiles are available in Supplementary Table S1 and have been previously described [7,8]. Feed intake was individually monitored using a Calan head-gate system (American Calan, Northwood, NH, USA), and rations were adjusted throughout pregnancy to support a target average daily gain of 0.45 kg per heifer through day 200. From day 201, feed was provided *ad libitum* to accommodate increased demands leading up to parturition. From day 240 onward, individual feed intake was recorded using the Insentec feeding system (Hokofarm Group B.V., Marknesse, The Netherlands). At birth, calves were immediately separated from their dams before suckling and housed individually. Within two hours of delivery, each calf received 1.4 L of commercial colostrum replacer (LifeLine Rescue High Level Colostrum Replacer, APC, Ankeny, IA, USA) administered via the esophageal feeder. Follow-up feedings of 2 L of milk replacer (Duralife 20/20 Optimal Non-Medicated Milk Replacer, Fort Worth, TX, USA) were given at 12 and 24 h after the initial colostrum supplement using the same method. At 30 h post-colostrum, euthanasia via captive-bolt stunning followed by exsanguination was performed in 14 calves (*n* = 7 per treatment group, female only). Organs were collected and weighed, and samples from the caudal to the midline position of the liver tissue were obtained, snap-frozen on dry ice, and stored at −80 °C.

### 2.3. RNA Extraction, RNA Sequencing, and Data Analyses

Liver tissue samples (30 mg) from 14 calves (*n* = 7 per group) were used for total RNA isolation, which was carried out using the RNeasy Kit (Qiagen, Germantown, MA, USA) in conjunction with an on-column DNase digestion step, as per the manufacturer’s guidelines. RNA integrity and purity were evaluated using a Qubit RNA IQ Assay kit (ThermoFisher Scientific, Carlsbad, CA, USA), agarose gel electrophoresis, and the Agilent 2100 Bioanalyzer (Agilent Technologies, Wilmington, DE, USA). All samples passed quality control thresholds for downstream processing. Strand-specific libraries were then constructed using the NEBNext^®^ Ultra™ II Directional RNA Library Prep Kit for Illumina (New England BioLabs^®^, Ipswich, MA, USA).

Sequencing was performed by Novogene Co., Ltd. (Nanjing, China) on the Illumina^®^ NovaSeq X platform. We generated 150 bp paired-end reads, targeting, on average, a sequencing depth of 20 million reads per library. Our data analyses followed the pipeline described in our previously published work on the transcriptome profile of jejunal mucosa [24] and shortly described on the following sections. Adapters and low-quality reads (Phred-Score < 30) were filtered out. FastQC v. 0.11.9 [25] and MultiQC v. 1.10.1 [26] were used for data quality control and read statistics evaluation. Quality parameters included average read length, adapter content, over-represented sequences, and per-base sequence quality scores. The STAR aligner v. 2.7.5 was used for mapping the reads to the *Bos taurus* reference genome (ARS-UCD1.2, Ensembl) [27]. To obtain the raw counts per gene, we used the –quantMode GeneCounts flag from STAR and the GTF annotation file from the Ensembl database (release 109). Post-mapping quality control was also performed using MultiQC. Following quality control, samples (*n* = 2, one from each group) with low unique mapping rates (% unique Aligned ≤ 80%) were removed from subsequent analyses.

### 2.4. Differential Expression Analysis

We used the *filterByExpr* function from edgeR v. 3.36.0 [28] to remove genes that were either not expressed or lowly expressed. Next, potential batch effects were investigated based on a principal component analysis implemented using the factoextra v1.0.7 [29] R-package. Differentially expressed genes (DEGs) were determined by comparing the VTM and CON groups using DESeq2 v.1.34.0 [30]. Significant differential expression was based on a *p* ≤ 0.05 and |log2 fold change| ≥ 0.5. The sign of the log2FC was used to classify the DEGs as up or downregulated in the VTM group. The DEGs were annotated using BiomaRt v. 2.50.1 [31].

### 2.5. Co-Expression and Regulatory Network Analyses

To explore regulatory mechanisms driving differential gene expression between the groups, we employed the Regulatory Impact Factor (RIF) analytical framework, utilizing both RIF1 and RIF2 algorithms [32]. RIF1 prioritizes transcription factors (TFs) that show distinct co-expression with highly expressed and differentially regulated genes, whereas RIF2 highlights TFs whose expression best predicts changes in the abundance of DEGs [32,33]. Accordingly, normalization of gene expression was performed using the *VST* function from DESeq2. A list of bovine TFs (*n* = 1445) was retrieved from the AnimalTFDB database v4.0 [34]. These TFs overlapped with our list of genes expressed in the liver, and those not expressed were filtered out (*n* = 483). The RIF analysis was performed using FORTRAN 90, following the published code provided by the authors [32]. Significant TFs were identified based on RIF1 or RIF2 values exceeding |1.96| standard deviations from the mean (*p* ≤ 0.05) [32,35].

To further characterize regulatory interactions, we applied the Partial Correlation and Information Theory (PCIT) algorithm to evaluate gene interaction patterns and differences in network connectivity between VTM and CON groups [36]. Networks were built separately for VTM and CON groups. Significant co-expression pairs were retrieved when a partial correlation ≥ |0.9| (*p* ≤ 0.05), and the correlated pairs were DEG or RIF genes [35].

Differences in network connectivity between groups were measured based on the differential connectivity, as follows: ${DK}_{i}=K_{VTM}\left(i\right)-K_{CON}\left(i\right),$ where *K* is the standardized connectivity for each gene in each network. The connectivity (K) was determined by normalizing each gene’s connectivity to the network’s maximum value [37]. Then, each *DK* score was transformed into a *z-score*. Scores were considered significant when values fell ± 1.96 standard deviations (SD) from the mean (*p* ≤ 0.05) [35]. Regulatory networks were visualized using Cytoscape (v3.9.0) [38].

### 2.6. Functional Over-Representation Analysis

To explore the effects of maternal diet on pathways and biological processes (BPs), we adopted two complementary approaches: over-representation (ORA) and gene set enrichment Analyses (GSEA). The ORA was performed using the DEGs list only, as we aimed to understand the BPs and KEGG pathways in which these DEGs were involved. To this end, we used ShinyGO v.0.80 [39] and ClueGo v2.5.10 [40], which integrate annotations from multiple databases and provide complementary visual and functional interpretations of enrichment results. Significance was determined based on the enrichment FDR ≤ 0.05 and Group *p*-value ≤ 0.05, respectively.

### 2.7. Gene Set Enrichment Analysis

To identify gene sets that share common biological pathways, while accounting for the collective effect of genes within a pathway [41], we performed GSEA using all expressed genes kept after post-mapping quality control. This complementary strategy was chosen because GSEA increases sensitivity to subtle but coordinated changes in gene expression that may not be captured by ORA alone [41]. The DESeqResults object from DESeq2 analysis was used to rank the genes, according to the following equation: $rank=\left[sign\left({log}_{2}FC\right)\times -{log}_{10}\left(p\text{-}\mathrm{Value}\right)\right]$, where the sign of the ranking (positive or negative) was based on the fold-change, while the magnitude was defined by the *p*-value [42].

The ranked gene list was used to categorize genes into KEGG pathways on the WebGestalt (WEB-based Gene SeT AnaLysis Toolkit v.2019) [43]. Under the assumptions of GSEA, the degree of over-representation is quantified using the normalized enrichment score (NES). The NES represents the enrichment score of a gene set after normalization across all analyzed gene sets. A significantly positive NES suggests that genes within the set are predominantly found at the top of the ranked list (positive FC), whereas a significantly negative NES indicates enrichment at the bottom of the list (negative FC) [41].

## 3. Results

### 3.1. Impact of VTM Supplementation on Maternal and Fetal Phenotypes

The experimental model and phenotypic results from this study were previously published [8]. Key findings were summarized here to provide context for the current molecular analyses. No significant differences were observed for heifer BW throughout gestation (CON: 510.1 ± 57.99 kg; VTM: 528.0 ± 65.86 kg; *p* ≥ 0.25), as well as at calving [8]. Similarly, VTM supplementation did not affect calf weights at calving or 30 h after [8]. No effects were identified in calf morphometrics and organ mass between treatment groups [8]. Regarding trace mineral status, dam serum concentrations at calving were similar across treatments, except for Co [8]. Calves from VTM supplemented cows had higher hepatic concentrations of Se and Mo, as well as Co and Se in the serum at 30 h of age [8].

### 3.2. Maternal Supplementation Alters Neonatal Liver Gene Expression and Transcription Factor Rewiring

The liver transcriptome profiles of neonatal heifer calves, taken 30 h after birth, were examined to determine differences based on maternal diets (VTM or CON) during gestation. Two samples were excluded from the study due to low alignment scores (below 80%). On average, 21.6 M clean reads per sample were produced through RNA-Sequencing. The minimum and maximum number of reads per sample ranged from 20.0 to 23.8 M, respectively. On average, 94.2% of all clean reads were uniquely aligned to the reference genome (Supplementary Table S2). After quality control of gene counts, 13,295 out of 27,607 genes remained in the 12 samples (*n* = 6 per group) for differential expression analysis.

**Figure 1:**
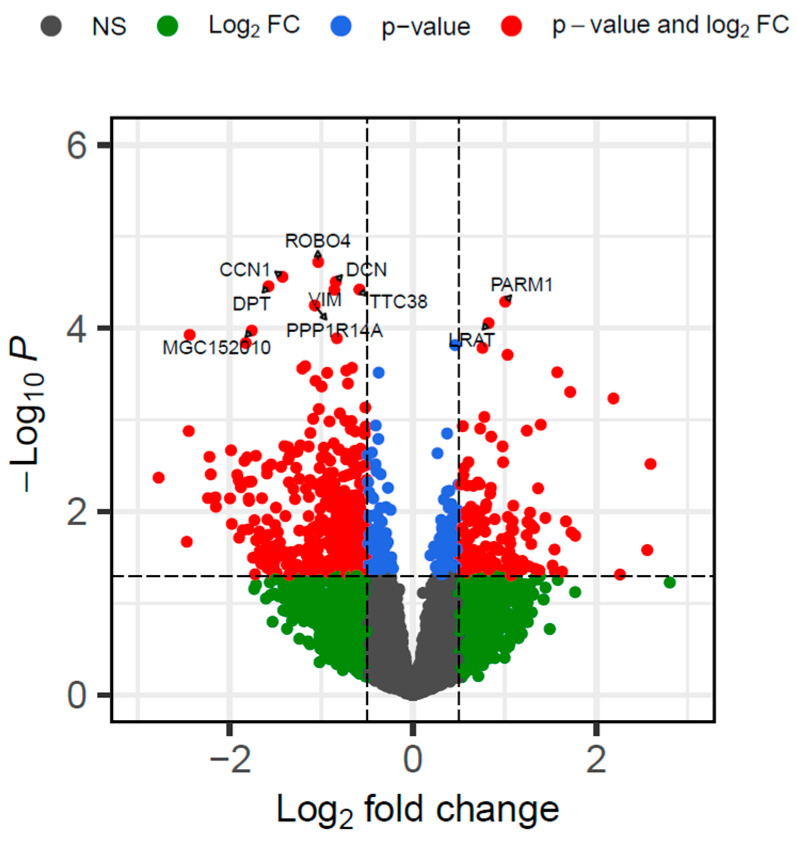
Volcano plot of differentially expressed genes in the liver tissue of newborn beef heifers, comparing offspring of VTM supplemented and control dams throughout gestation. Each dot represents a gene. Only the top 10 genes are labeled. The x-axis shows the log2 fold change differences, whereas the y-axis shows the log (base 10) of the *p*-value. Genes in red are up or downregulated in the VTM group. The horizontal dashed line represents the selected significance threshold (*p* ≤ 0.05), whereas the vertical dashed line corresponds to the upregulated and downregulated fold change thresholds (log2FC| ≥ 0.5).

Differential expression analyses using DESeq2 identified 630 DEGs by comparing the VTM and CON groups (*p* ≤ 0.05 and |log2FC| ≥ 0.5). Of these, 160 genes were upregulated and 470 were downregulated in the calves born from VTM cows (Figure 1). Protein-coding genes accounted for the majority of DEGs (94.1%); however, long non-coding and small non-coding RNAs were also identified (Supplementary Table S3). The top five DEGs with the most significant *p*-values included *ROBO4*, *CCN1*, *DCN*, *DPT*, and *TTC38.* Among the DEGs, we identified 36 TFs, including *CREB3L1*, *STAT5A*, and *FOS*.

We used the RIF metrics to identify TFs potentially modulating the expression of DEGs. We identified 58 significant TFs (*p* ≤ 0.05; RIF1 = 55; RIF2 = 3) out of 962 tested as potential regulators. These TFs were classified into 19 families, with the zinc finger C2H2 and homeobox families being over-represented. The TFs *ZNF133* (*z-score* = 3.98), *GRHL2* (*z-score* = 3.61), *NFIC* (*z-score* = 3.3), *FOXQ1* (*z-score* = 3.15), and *MKX* (*z-score* = 3.09) showed the most extreme scores for RIF1. For RIF2, the list included *CIZ1* (*z-score* = 2.106), *HSF1* (*z-score* = 2.01), and *MTA1* (*z-score* = 1.96). A list of all RIF1 and RIF2 significant TFs is presented in Supplementary Table S4. Herein, genes encoding TFs, identified as key regulators based on the RIF score, will be called TFs throughout the text.

**Figure 2:**
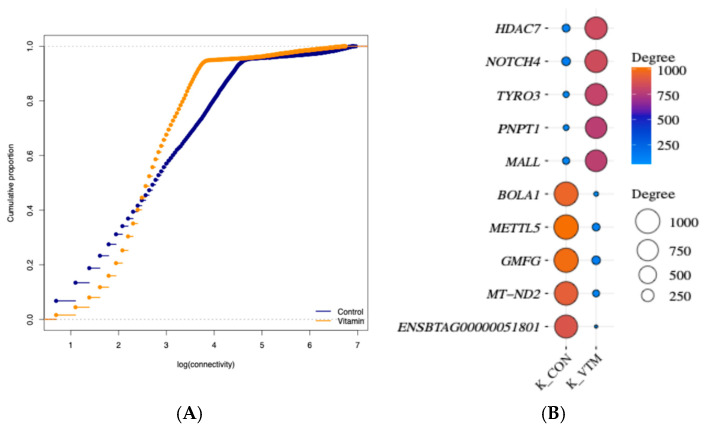
Topological features of gene co-expression networks in the liver tissue of newborn beef heifers, comparing offspring of VTM supplemented and control dams throughout gestation. (**A**): Cumulative distribution function plot of the gene connectivity of networks from VTM or CON groups. The x-axis represents the log of gene connectivity (the number of co-expression links each gene has within the network), and the y-axis represents the cumulative proportion of genes with connectivity less than or equal to a given value; (**B**): Balloon plot showing the five highest and lowest ranked differentially expressed and connected (*DK*) genes. Differential connectivity represents the variation in the number of network connections a gene has across the two groups, providing insight into how supplementation alters gene–gene relationships. Gene names are shown on the y-axis, and the connectivity for CON and VTM groups is shown on the x-axis. The size and color of the balloon refer to the total number of connected genes in each group. The plot was created using SRplot [44].

We conducted a network analysis based on the PCIT approach [36] to explore the gene co-expression patterns and identify the most rewired ones between the groups in response to maternal diet. The networks from the CON and VTM groups resulted in 2,717,766 and 2,440,509 gene pairs, respectively. Data dimensionality was reduced by retaining only co-expressed pairs with DEGs or RIF genes and a partial correlation ≥ |0.9| (*p* ≤ 0.05). The CON network resulted in 302,398 gene pairs (12,787 unique genes), while for the VTM, we identified 196,459 gene pairs (13,238 unique genes). However, only 12,725 genes were shared between both networks, and 516 were unique to the VTM network. Through comparison of connectivity across groups, we found 435 differentially connected (*DK*) genes (*p* ≤ 0.05; Supplementary Table S5). Although the VTM group showed an increase in the number of unique genes within the network, the number of connections was reduced, and 274 genes lost connectivity in the subnetwork based on a correlation greater than |0.9| (Figure 2). Among the 43 TFs differentially connected, 26 TFs gained connectivity in the VTM network, whereas 17 TFs lost connectivity. The TFs with the highest *DK* included *ZNF200* (*z-score* = 7.9), *ZNF397* (*z-score* = 7.1), and *ZFP62* (*z-score* = 7.0), while the DEGs also differentially connected were *HDAC7* (*z-score* = 9.9), *NOTCH4* (*z-score* = 9.9), and *TYRO3* (*z-score* = 9.8) (Figure 2).

### 3.3. Differentially Expressed Genes Were Involved with Oxidative Phosphorylation, Vitamin Digestion and Absorption, and Metabolism Processes

**Figure 3:**
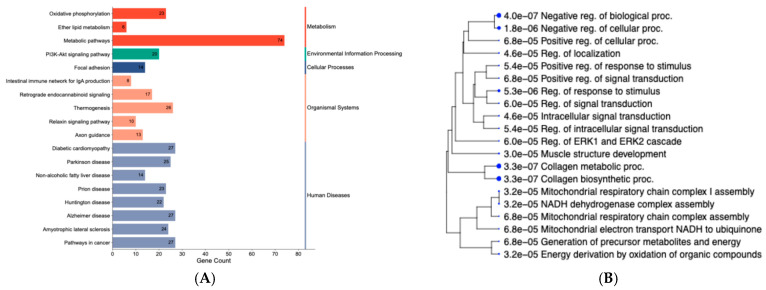
KEGG pathways and gene ontology terms based on differentially expressed genes in the liver tissue of newborn beef heifers, comparing offspring of VTM supplemented and control dams throughout gestation. (**A**): Enrichment category plot of classified KEGG pathways based on the DEG list. The plot was created using SRplot [44]. The pathways were further classified into five major groups: metabolism, environmental information processing, cellular processes, organismal systems, and human diseases; (**B**): Hierarchical clustering of over-represented biological processes (BPs). The BPs are organized hierarchically according to their functional similarity. Dot size corresponds to the statistical significance of each term (FDR ≤ 0.05).

A two-tiered approach was taken to identify BPs and KEGG pathways related to changes in gene expression. First, the over-representation analysis of DEGs (*n* = 630) retrieved the top 20 BPs and 20 KEGG pathways (Figure 3A; Supplementary Table S6). KEGG pathways included oxidative phosphorylation, non-alcoholic fatty liver disease, PI3K-Akt signaling pathway, and metabolic pathways (Figure 3A). Shared genes within the pathways were in the COX protein complex family (*COX1*, *COX2*, *COX3*), NADH-ubiquinone oxidoreductase chain *(ND1*, *ND2*, *ND3*, *ND4*, *ND4L*, *ND5*, *ND6)*, and the NADH dehydrogenase [ubiquinone] 1 alpha subcomplex (*NDUFA2*, *NDUFA3*, *NDUFA8*, *NDUFA11*, *NDUFA4L2*). Additionally, over-represented pathways from the ClueGo analysis (Supplementary Table S6), considering the 630 DEGs, included FoxO signaling, TGF-beta signaling, and complement and coagulation cascades (Group *p*-value ≤ 0.05). Significant BPs retrieved from ShinyGO were related to the mitochondrial respiratory chain, metabolic processes, and signal transduction (Figure 3B). Furthermore, BPs related to tissue structure and development included collagen biosynthetic processes and collagen metabolic processes. Among the underlying genes shared across BPs, we can highlight *BMP4*, *COL1A1*, *F2R*, *RCN3*, *CREB3L1*, and *TGFB3*.

**Figure 4:**
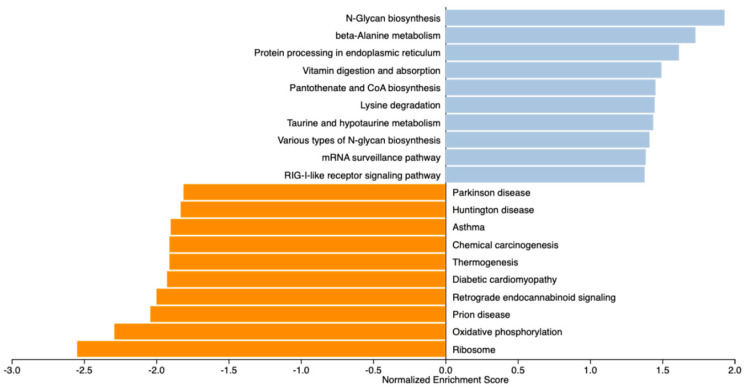
Gene Set Enrichment Analysis (GSEA) of gene profiles in the liver of newborn beef heifers, comparing offspring of VTM supplemented and control dams throughout gestation. Pathways are ranked considering concordant differences in gene expression (*n* = 13,295) between VTM and CON groups. Normalized enrichment score (NES) indicates the degree to which a pathway is enriched at the top or bottom of the ranked gene list after adjusting for gene set size within the dataset. Positive NES scores (blue bars) indicate pathways enriched in VTM calves, while negative NES scores (orange bars) indicate pathways depleted in VTM calves. Significant pathways were determined based on *p* ≤ 0.1 and |NES| ≥ 1.5.

To gain a more comprehensive understanding of the pathways influenced by the maternal treatments, we conducted a GSEA analysis on 13,295 expressed genes. After ranking all the genes, we identified the top 20 KEGG pathways (|NES ≥ 1.5|). The top pathways involving positively ranked genes included beta-alanine metabolism, vitamin digestion and absorption (Figure 4). *ALDH7A1*, *SLC19A3*, *LMBRD1*, *JARID2*, *ACVR1B*, *PNPO*, *TRADD*, and *TBK1* were underlying the over-represented pathways.

**Figure 5:**
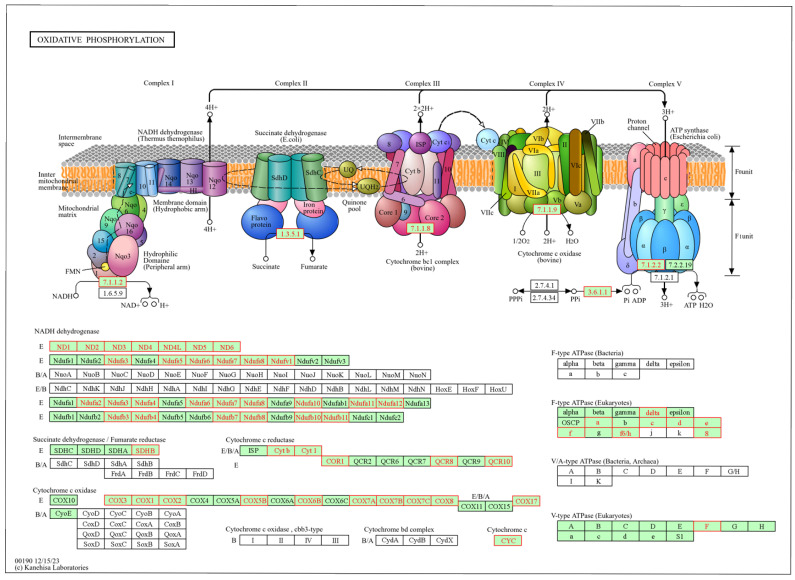
Oxidative phosphorylation metabolic pathway identified from the gene set enrichment analysis (GSEA) based on genes in the liver of newborn beef heifers, comparing offspring of VTM supplemented and control dams during gestation. Green boxes with red borders represent negatively ranked genes based on the VTM vs. CON comparison underlying the pathway. Pathway from the Kanehisa Laboratories [45].

Conversely, the top identified pathways underlying the negatively ranked genes included ribosome and oxidative phosphorylation (Figure 5), over-represented by downregulated DEGs from the COX protein complex, NADH-ubiquinone oxidoreductase chain, and the NADH dehydrogenase [ubiquinone] 1 alpha subcomplex. Shared pathways between GSEA and KEGG analyses identified with ClueGO and ShinyGO were the intestinal immune network for IgA production, retrograde endocannabinoid signaling, and oxidative phosphorylation.

## 4. Discussion

We investigated the effects of maternal vitamin and mineral supplementation during pregnancy on the gene profile of neonatal liver tissue. Our results show that maternal supplementation affected the expression profile of genes playing key roles in liver metabolism and function. Furthermore, we shed light on the regulatory interplay between genes and TFs and their rewiring, leading to differences in the transcriptional regulation between the VTM and CON groups.

Our previous findings provided strong evidence that maternal vitamin and mineral supplementation from the periconceptual period to day 83 of gestation affected maternal and fetal development [5,6,10,20]. We have reported increased concentrations of Se, Cu, Mn, and Co in the liver and amino acids in allantoic fluid from the VTM group [46]. Fetuses from VTM dams had heavier livers and altered metabolome and transcriptome, which were over-represented by lipid and metabolism-related processes [5,6,20]. In the current study, where supplementation extended throughout gestation, we did not identify any treatment effect on BW at calving or organ mass [8]. These differences may be due to fetal developmental plasticity and maternal buffer effects, where compensatory mechanisms may normalize growth trajectories throughout gestation [47]. On the other hand, the concentration of key trace minerals in the serum (Co, Se, and Zn) and liver (Se) of the calves was affected by the maternal diet [8]. In a similar study with a different cohort, we reported that F1 female heifers born to VTM dams were significantly heavier from weaning through 15 months of age, although no differences in birth weight were reported [7]. Harvey et al. [48] found that supplementation of organic-complexed or inorganic Co, Cu, Mn, and Zn to cows during the second and third trimesters of gestation had no effect on calf liver trace mineral concentrations at birth, 24 h after, and at weaning. Additionally, no effects were observed on calf birth weight and weaning BW. Conversely, supplementation during the last trimester of gestation, with organic or inorganic Co, Cu, Mn, and Zn, affected the concentrations of hepatic Co, Cu, and Zn. Although no differences were reported for BW at birth, calves born to cows supplemented with organic complexed minerals were heavier from weaning until slaughter [48]. While phenotypic effects may be timing, duration, and nutrient-dependent, our results suggest molecular programming with phenotypic responses that may be delayed or latent and only emerge later in life or under specific environmental stimuli [49].

### 4.1. Maternal Vitamin and Mineral Supplementation Affected Neonatal Gene Expression and Rewired Key Regulatory Genes Underlying Hepatic Metabolism

Vitamins and minerals are key structural or co-factor components of catalytic enzymes and TFs [50]. Additionally, they can modulate the genome through nutrient response elements and epigenetic mechanisms [51]. Imbalances in nutrient availability during key developmental windows may induce responsive and adaptive changes in development through TFs modulating differential gene expression [14]. We identified 36 TFs among the 630 DEGs between the VTM and CON groups. Key TFs involved with liver metabolism included *STAT5A* and *CREB3L1* genes. The *STAT5A* gene, which was downregulated in the VTM group, is a transcriptional activator of metabolic genes associated with physiological processes such as body growth, lipid, bile acid, and steroid metabolism [52]. STAT5A activation in the liver is a key pathway in response to GH signaling, which in turn activates the transcription of the *IGF1* gene [52]. The concentration of GH metabolites tended to be significantly higher in the serum of dams from the VTM group [8]. For the calves, the serum concentration of GH and IGF1 metabolites was not significantly different between groups [8]; however, the *IGF1* gene was upregulated in the calves born from VTM dams. Minerals, such as Mg, Se, and Zn, have been associated with IGF1 levels [53]. We have shown that fetuses born to VTM supplemented dams during the first trimester of gestation had higher concentrations of Se in the liver [8]. The modulation and bioavailability of IGF1 are complex due to its interplay with insulin-like growth factor binding proteins (IGFBPs) and IGF1 receptors (IGF-1R) [54]. In the current study, we identified the *IGFBP7* and *IGFBP6* genes downregulated in the VTM group, which may suggest reduced inhibition of IGF1 activity [54].

The *CREB3L1* gene is a TF activator involved in endoplasmic reticulum stress response and secretory pathway regulation [55]. As a nutrient-responsive TF, CREB3L1 interacts with PPAR alpha and FOXO1 to regulate energy homeostasis via lipid and glucose metabolism [55,56]. PPAR protein plays an adaptive role in tissue development in response to nutrient availability by forming transcriptional complexes with nuclear receptors that activate fatty acid oxidation and insulin secretion genes [14,57]. We identified the *IRS2* and *BMP7* genes upregulated, and the *BMP4* downregulated in the VTM group. *IRS2* encodes a protein associated with insulin signaling pathways and serves as an adapter protein that activates insulin and IGF1 receptors to downstream signaling molecules, such as PI3K and Akt [58]. The BMP7 regulates insulin sensitivity by inhibiting MAPKs [59], whereas BMP4 promotes hepatic glycogen accumulation [60]. The *IGF1* and *CREB3L1* genes, among other DEGs, were underlying nutrient-sensing pathways. The PI3K-Akt, AMPK, and FoxO pathways are interconnected to maintain energy homeostasis [61,62].

The regulatory role of the genes was perturbed due to maternal diet, as evidenced by the 435 differentially connected genes we identified. The TFs identified as RIFs were rewired, and most gained connections (*n* = 26), suggesting that they differentially modulated gene transcription between the groups. The top four TFs that gained connections in the network from the VTM group were members of the zinc finger protein family (*KLF16*, *ZFP62*, *ZNF397*, and *ZNF200*). This gene family plays key roles in tissue homeostasis and has been associated with pathological conditions related to liver disease in humans [63]. The regulatory mechanisms and specific role of these TFs, however, are yet to be elucidated.

Zinc finger proteins interact with modified histones, suggesting a role in epigenetic regulation. Ocampo et al. [64] reported that Zn status alters chromatin accessibility, affecting distal regulatory regions and promoters. In vitro studies with murine cells have shown epigenetic modulation of proinflammatory genes through histone acetylation in response to selenite supplementation [65]. Histone deacetylation modulates gene expression by removing acetyl groups from histones and TFs, leading to transcriptional repression [66]. We identified histone deacetylating genes, *HDAC4*, *HDAC6*, and *HDAC7*, among the DEGs. While the first was upregulated, *HDAC6* and *HDAC7* were downregulated in the VTM group. Decreased *HDAC4* has been associated with decreased proinflammatory gene expression, insulin resistance, and obesity [67]. Interestingly, both *HDAC6* and *HDAC7* were more connected to the VTM group, suggesting increased chromatin accessibility for transcription. Epigenetic mechanisms, such as DNA methylation, histone methylation, and acetylation, are affected by mineral imbalances [4]. We have shown that fetuses from VTM supplemented dams at day 83 of gestation had 60% hypermethylated cytosines in the liver, potentially regulating 739 nearby genes [68]. Biological processes affected by these genes included the transport of ions and tissue structure development [68]. These findings suggest changes in genes underlying tissue structure and function through epigenetic mechanisms, which may contribute to offspring metabolic programming [16].

### 4.2. The Vitamin Digestion and Absorption Pathway Is Over-Represented by Positively Ranked Genes

Serum concentrations of vitamin D were increased in supplemented dams and their calves. In contrast, vitamin A concentrations were increased only in the dams compared to the CON counterparts [8]. However, vitamin A is poorly transferred across the placenta, which may have limited its availability to the developing fetus [69]. Despite that, calves born to VTM cows showed the *LRAT* gene upregulated. The protein coded by *LRAT* is an essential component of vitamin A metabolism [70]. Retinoic acid and calcitriol influence gene expression by direct interaction with regulatory elements [71]. The nuclear receptors RAR and RXR are activated by retinoic acid via binding to retinoic acid response elements, which induce or repress transcription of targeted genes [50,51]. Harris et al. [72] reported that administering a vitamin A injection to neonatal calves increased weaning weight and enhanced marbling fat development in Angus steers. On the other hand, vitamin A injection into pregnant Nellore cows at 250 days of gestation positively impacted average daily gain before weaning and final BW in the finishing phase in steers [73]. Besides *LRAT*, the *SLC19A2*, *SLC19A3*, *ABCC1*, *LMBRD1*, and *BTD* genes were over-represented in the vitamin digestion and absorption pathway retrieved from the GSEA analysis. These genes were positively ranked, which suggests they were upregulated in a coordinated way. *SLC19A2* and *SLC19A3* code a thiamine and pyridoxine transmembrane transporter (vitamins B1 and B6) [74], whereas the *BTD* gene codes for biotinidase, which recycles biotin (vitamin B7). These proteins serve as enzymatic co-factors in energy metabolism. For example, biotin acts as a co-factor of gluconeogenic enzymes pyruvate carboxylase (PC) and propionyl-coenzyme A carboxylase (PCC) [75].

Genes associated with vitamin B5 (*PANK1*, *PANK3*, *VNN2*, *ENPP1*, *CSAD*, and *PPCS*) were positively ranked in our GSEA analysis, underlying the pantothenate and CoA biosynthesis pathway. The *PANK* genes are members of the pantothenate kinase family, which are key regulatory enzymes in the biosynthesis of coenzyme A (CoA) [76]. The *PANK1* gene, which was upregulated in the VTM group, together with *PANK3*, catalyzes the first committed step and controls the overall rate of CoA biosynthesis [77]. From the same pathway, the gene *PPCS* catalyzes the second step in the biosynthesis of CoA [78]. B vitamins are usually not supplemented in beef production systems, as ruminal microbes provide a substantial supply of most water-soluble vitamins [79].

Trace minerals play key roles in the synthesis of B vitamins by acting as co-factors for microbial enzymes [80]. Among the minerals, Co is used for the synthesis of vitamin B12 [80]. The protein coded by the gene *LMBRD1*, associated with the vitamin digestion and absorption pathway, mediates the transport and metabolism of vitamin B12. This vitamin plays a key role in the metabolism of branched-chain amino acids and odd-chain fatty acids [81,82]. We have reported increased hepatic storage of Co throughout gestation, and VTM supplemented dams had greater Co concentration in total colostrum [7]. In the current study, VTM supplemented dams and their calves had greater Co concentrations in the serum at calving [8]. As neonatal calves do not have a functional rumen and established microbial community, they rely on placental and colostral transfer of B vitamins to meet their requirements [83]. Therefore, calves born to supplemented dams likely had greater B vitamin concentrations at birth. Duplessis and Girard [83], reported that maternal biotin, folic acid, and vitamin B12 supplementation 26 days before calving increased dam plasma and colostrum vitamin and calf plasma folate concentrations. These findings indicate that maternal VTM supplementation positively affects vitamin-related metabolic pathways in the calf, potentially supporting early postnatal vitamin utilization and metabolism.

### 4.3. The Oxidative Phosphorylation Pathway Was Over-Represented by Downregulated Genes in the Liver of Calves Born to VTM Supplemented Dams

Oxidative phosphorylation (OXPHOS) is the primary pathway by which cells generate ATP from the breakdown of carbohydrates and fats to provide cellular energy [84]. This process takes place in the mitochondria through a series of redox reactions that drive the electron transport chain and ATP synthase activity [85]. We identified 23 DEGs that were all downregulated in the liver of VTM supplemented calves and associated with the OXPHOS KEGG pathway. Curiously, all of them lost connectivity within the VTM network group. In the same pathway, an additional 34 genes were negatively ranked within the GSEA analysis. Among them were genes that are part of the NADH dehydrogenase (ubiquinone) complex I gene family. These genes regulate mitochondrial complex I activity by transferring electrons from NADH to the respiratory chain [86]. Also, we identified the mitochondrial genes *MT-ND6*, *MT-ND4L*, *MT-ND3*, *MT-ND2*, and *MT-ND4*, which are part of the complex I assembly.

Micronutrients play significant roles in mitochondrial function, particularly in energy metabolism and ATP production, as many serve as essential co-factors. Notably, 11 of the 12 minerals required for human health are directly involved in mitochondrial metabolic processes [87]. The availability of trace minerals influences the assembly, stability, and activity of the electron transport chain [88]. However, the excess availability of redox-active co-factors may contribute to the downregulation of genes involved in the electron transport chain as a regulatory mechanism to mitigate the potential overproduction of reactive oxygen species, which are closely associated with mitochondrial Complex I activity [89]. Therefore, the observed downregulation of Complex I genes despite increased mineral availability may reflect a feedback or adaptive response to maintain mitochondrial redox homeostasis under enhanced micronutrient conditions. The relative downregulation of the above-mentioned genes in the VTM group means an upregulation in the CON counterpart. These changes may be related to a compensatory activation, such as beta-oxidation, to be used as the main energy source [90]. Additionally, if required co-factors are limited, it is possible that cells may transcriptionally upregulate genes involved in oxidative phosphorylation to maximize efficiency of energy generation and compensate for potential reductions in enzymatic activity.

The hepatic mitochondrial function was previously assessed via high-resolution respirometry using the same cohort of calves we used in this study. No significant differences were observed in LEAK and OXPHOS respiration parameters between treatments, whereas there was a significant increase in residual oxygen consumption (ROX) and a trend for increased electron transport system capacity (E) in the VTM group [46]. Despite unchanged whole-tissue OXPHOS function at the neonatal stage, transcriptomic changes suggest reduced ATP production and functional reprogramming of mitochondrial pathways in VTM calves. These findings may support the hypothesis of compensatory effects due to the upregulation of the OXPHOS genes in the CON group to coordinate energy metabolism. In mice, the loss of mitochondrial complex I has been shown not to impair the liver’s ability to maintain metabolically functional mitochondria [91]. Although not over-represented in the OXPHOS pathway, we identified the *MTA1* gene as significantly less connected in the VTM group. This gene has been reported as part of the deacetylation complex [92]. Additionally, *MTA1* is an ATP synthase modulator, driving mitochondrial bioenergetic metabolism reprogramming associated with growth and liver metastasis of colon cancer [93]. These results corroborate our previous findings in the jejunal mucosa of the same calves. We have reported changes in the expression of nutrient transporter-related genes, metabolism, tissue structure, and immune function [24]. While these findings may reflect early-life adaptations that could influence energy metabolism, further studies are needed to determine potential regulatory mechanisms and functional metabolic outcomes.

When considered together, findings from our series of studies highlight the importance of maternal vitamin and mineral supplementation throughout gestation. Supplementation from approximately day-60 pre-breeding to day 83 of gestation altered fetal liver mass, gene expression, and the metabolome [5,6,20]. Similarly, key hepatic genes were affected at birth when supplementation was provided until calving (current study). Moreover, supplementation of dams from breeding to calving resulted in phenotypic improvements, including greater body weight of F1 heifers at 15 months of age [7]. The results of the current experiment specifically isolate the impacts of gestational nutrition on fetal liver programming. Our previous experiments indicate that liver gene expression was influenced during early gestation [6], but left unanswered questions regarding the effects across the full course of gestation and the postnatal consequences. Additional work using a model in which calves were born and raised by their dams indicates that the postnatal growth phenotype was also affected [7]. However, these postnatal outcomes are confounded by the impacts of programming during gestation, and the postnatal impacts of the dam’s mothering ability, milk production, etc. Thus, the present study fills a critical gap by isolating gestation as the key interval, showing that supplementation with vitamins and minerals during this period results in calves born with altered hepatic transcripts affecting important metabolic pathways. Although birth weight differences were not consistently observed in previous models, the transcriptomic alterations reported here suggest potential programming effects that may influence postnatal performance, as pointed out by Hulbert et al. [7]. These results demonstrate that maternal supplementation influences both molecular programming and offspring performance, highlighting its practical relevance for producers. Therefore, providing a balanced vitamin and mineral premix is a management strategy that can potentially improve calf development, growth, and long-term productivity in beef production systems.

The results of the current study should be interpreted considering the limited sample size used for gene expression analysis. Additionally, our study was restricted to a single time point and relied on transcriptomic profiling and high-resolution mitochondrial respirometry in the jejunal mucosa and liver tissues. While this provides insights into the nutrient-induced programming, it does not fully capture the complexity of regulatory mechanisms underlying tissue programming. Complementary assays at the protein or metabolite level and longitudinal sampling at postnatal stages would further strengthen the findings reported. Finally, because we have used a commercially available vitamin-mineral premix, it is not possible to disentangle the specific contributions of individual nutrients. However, this approach reflects industry practice and enhances the real-world applicability of our findings. Further analyses should focus on epigenetic mechanisms, including DNA methylation, chromatin modification, and miRNA regulation. While the expression of a single gene is unlikely to drive a complete physiological shift, the differential expression patterns and regulatory network rewiring observed within key metabolic pathways suggest potential functional adaptations due to maternal diet. Longitudinal studies encompassing various prenatal and postnatal stages would provide a more comprehensive understanding of the long-term implications of short-term feeding decisions. In a similar study using a different cohort, we reported that F1 heifers born to VTM dams have enhanced post-weaning development [7], suggesting that programming effects may remain latent [49]. Further research should explore whether epigenetic marks are established at birth, persist throughout postnatal development, and potentially contribute to multigenerational inheritance.

## 5. Conclusions

Our findings support the hypothesis that maternal vitamin and mineral supplementation throughout gestation is associated with molecular changes in the neonatal liver via transcriptomic responses. We identified differences in the wiring of key TFs involved with liver function and metabolism. Overall, networks from the VTM group were rewired and lost connections, although most TFs increased their connectivity. In particular, VTM supplementation led to differential regulation of histone deacetylating genes (*HDAC4*, *HDAC6*, and *HDAC7*). Further research is warranted to determine the molecular mechanisms by which maternal vitamin and mineral supplementation programs offspring liver function. Targeted epigenomic research will help to determine how epigenetic mechanisms, such as DNA methylation and chromatin remodeling, contribute to nutrient-induced programming. In addition, long-term and multigenerational studies are necessary to assess whether maternal supplementation influences the growth, health, and productivity of offspring beyond the neonatal stage. Finally, evaluating practical supplementation strategies in production settings will be important to identify optimal formulations, timing, and delivery methods that maximize the potential benefits of fetal programming for cow-calf producers.

## Data Availability

The data are within the paper and its Supplementary Information files. The RNA sequencing data can be accessed through NCBI GEO database (http://www.ncbi.nlm.nih.gov/geo/) with accession number GSE307455.

## Supplementary Materials

The following supporting information can be downloaded at https://www.mdpi.com/article/10.3390/ani15182664/s1: Supplementary Table S1: Nutrient composition of vitamin and mineral (VTM) supplement provided to beef heifers; Supplementary Table S2: Summary of RNA sequencing, mapping statistics, and treatments; Supplementary Table S3: Differentially expressed genes in the liver tissue of newborn beef heifers, comparing offspring of VTM supplemented and control dams during gestation; Supplementary Table S4: Transcription factors identified as potential modulators of differential expression in the liver tissue of newborn beef heifers, comparing offspring of VTM supplemented and control dams during gestation by Regulatory Impact Factor measures (RIF1 and RIF2); Supplementary Table S5: Differentially connected genes in the liver tissue of newborn beef heifers, comparing offspring of VTM supplemented and control dams) during gestation retrieved from PCIT analysis. (|r|≥ 0.9, *p* ≤ 0.05); Supplementary Table S6: Functional over-representation analysis of differentially expressed genes in the liver of newborn beef heifers, comparing offspring of VTM supplemented and control dams during gestation.

## Abbreviations

The following abbreviations are used in this manuscript:

BP	Biological processes
BW	Body weight
CON	Control
DEG	Differentially expressed genes
DK	Differential Connectivity
FC	Fold Change
GSEA	Gene Set Enrichment Analysis
log2FC	log 2-fold change
K	Connectivity
NES	Normalized Enrichment Score
PCIT	Partial Correlation and Information Theory
RIF	Regulatory Impact Factor
TF	Transcription Factors
VTM	Vitamin and mineral supplementation
